# Imeglimin-based therapies improve glycemic control and reduce mitochondrial stress in type 2 diabetes: a prospective cohort study

**DOI:** 10.3389/fendo.2025.1639046

**Published:** 2025-09-23

**Authors:** Abhishek Satheesan, Janardanan Subramonia Kumar, Leela Kakithakara Vajravelu, Ria Murugesan

**Affiliations:** ^1^ Department of Microbiology, SRM Medical College Hospital and Research Centre, Faculty of Medicine and Health Sciences, SRM Institute of Science and Technology, Kattankulathur, Chengalpattu, Tamil Nadu, India; ^2^ Department of General Medicine, SRM Medical College Hospital and Research Centre, Faculty of Medicine and Health Sciences, SRM Institute of Science and Technology, Kattankulathur, Chengalpattu, Tamil Nadu, India

**Keywords:** Imeglimin, type 2 diabetes mellitus, ccf-mtDNA, NLRP3 inflammasome, HbA1c, oral hypoglycemic agents

## Abstract

**Background:**

Imeglimin, a novel oral antidiabetic agent, has demonstrated mitochondrial and anti-inflammatory benefits. This study evaluated the efficacy of Imeglimin-based therapies on glycemic control, mitochondrial stress (circulating cell-free mitochondrial DNA (ccf-mtDNA), and inflammation in type 2 diabetes mellitus (T2DM).

**Methods:**

A total of 104 T2DM patients were enrolled and assigned to one of four groups out of which 96 patients completed follow-up and data was analyzed: Imeglimin monotherapy (n=23), Imeglimin + Metformin (n=24), Imeglimin + other Oral Hypoglycemic Agents (OHAs) (n=24), and Metformin + other OHAs (n=25). Assessments at baseline and 6 months included HbA1c, lipid profile, ccf-mtDNA, NOD-like receptor family, pyrin domain containing 3 (NLRP3), Interleukins-6, 1β and 18 (IL-6, IL-1β, and IL-18). Within-group changes were assessed using paired t-tests. Repeated measures ANCOVA models analyzed group-time interactions. Correlation analysis explored associations between Δ biomarkers and metabolic parameters.

**Results:**

Combination therapies, particularly Imeglimin + other OHAs, significantly reduced HbA1c (Δ=–0.5%, p=0.001), ccf-mtDNA (Δ=–18.5 copies/μL, p=0.02), and IL-6 (p< 0.001). Repeated measures ANCOVA revealed significant reductions in HbA1c (p=0.001), circulating cell-free mtDNA (p=0.004), and serum NLRP3 levels (p=0.037) across imeglimin-based therapy groups. *Post hoc* comparisons showed the greatest improvements in the Imeglimin + Other OHAs group versus control. Significant time × group effects for IL-6 and IL-1β. No changes were noted in IL-18.

**Conclusion:**

Imeglimin, especially in combination with non-Metformin OHAs, improves glycemic control and reduces mitochondrial and inflammatory stress in T2DM patients. These findings support its use as an adjunctive therapy with broader metabolic benefits.

**Clinical Trial Registration:**

https://ctri.nic.in/Clinicaltrials/advancesearchmain.php, identifier CTRI/2023/12/060844.

## 1 Introduction

Type 2 diabetes mellitus (T2DM) is a progressive metabolic disorder characterized by chronic hyperglycemia and insulin resistance, with systemic low-grade inflammation playing a central role in the pathophysiology and progression of its complications (1). Mitochondrial dysfunction and innate immune activation are increasingly recognized as key contributors to this inflammatory milieu. In particular, circulating cell-free mitochondrial DNA (ccf-mtDNA), a damage-associated molecular pattern (DAMP), can activate the NLRP3 inflammasome, a cytosolic protein complex responsible for the cleavage and secretion of interleukin (IL)-1β and IL-18, further amplifying inflammatory responses (2–4).

Imeglimin is a first-in-class oral antidiabetic agent belonging to the tetrahydrotriazine-containing compound family. Chemically, it is a small heteroaromatic molecule with a unique tetrahydrotriazine ring structure. While structurally distinct, it shares some similarities with metformin, as both are guanidine derivatives with a biguanide-like pharmacophore that contributes to their mitochondrial-targeted actions. This structural resemblance partly explains overlapping mechanisms, such as inhibition of mitochondrial complex I and modulation of hepatic glucose production. However, unlike metformin, Imeglimin exhibits a pleiotropic “double action” by not only improving insulin sensitivity but also directly enhancing pancreatic β-cell function and survival. Beyond its glycemic control, emerging evidence suggests its potential to attenuate mitochondrial-derived oxidative stress and inflammation. However, clinical data evaluating the impact of Imeglimin on key mitochondrial and inflammasome-related biomarkers in T2DM patients remain limited. In 2021, it became the first drug of its class to receive regulatory approval in Japan for the treatment of type 2 diabetes, following the TIMES clinical trial program (5).

Circulating cell-free mitochondrial DNA (ccf-mtDNA) is gaining attention as a sensitive indicator of mitochondrial dysfunction, particularly in metabolic diseases such as type 2 diabetes. Released into the bloodstream from damaged or stressed cells, ccf-mtDNA reflects disturbances in mitochondrial integrity and cellular homeostasis (6, 7). In diabetic individuals, elevated ccf-mtDNA levels are commonly associated with increased oxidative stress, impaired mitochondrial respiration, and systemic inflammation.m (8, 9) Circulating cell-free mitochondrial DNA (ccf-mtDNA) functions as a damage-associated molecular pattern (DAMP), capable of activating innate immune responses, including the NLRP3 inflammasome pathway. This activation promotes the release of pro-inflammatory cytokines, such as IL-1β and IL-18, which are implicated in sustaining the low-grade inflammation characteristic of diabetes (10–12). Therefore, measuring both ccf-mtDNA and serum NLRP3 levels can provide complementary insights into mitochondrial distress and inflammasome activation. A positive correlation between mtDNA and NLRP3 suggests that mitochondrial dysfunction may directly fuel inflammatory signaling in diabetes, offering potential targets for early detection and therapeutic intervention.

This prospective 6-month study aims to evaluate the effect of Imeglimin therapy on serum levels of ccf-mtDNA and NLRP3 inflammasome expression in patients with T2DM. Additionally, we investigate the association of these novel biomarkers with conventional inflammatory markers including and interleukins, to better understand the inflammatory modulation profile of Imeglimin and its potential therapeutic implications in diabetes-related inflammation.

## 2 Methodology

### 2.1 Study design and participants

This was a prospective observational study conducted to evaluate the effect of Imeglimin therapy on circulating cell-free mitochondrial DNA (ccf-mtDNA) levels and NLRP3 inflammasome expression in patients with type 2 diabetes mellitus (T2DM), and to assess their association with conventional inflammatory markers. A total of 104 T2DM patients were enrolled from the Diabetes Outpatient Clinic, Department of General Medicine, SRM Medical College Hospital and Research Centre, Kattankulathur, India.

The sample size calculation was based on anticipated effect sizes for the primary outcome (HbA1c) and a key secondary biomarker (mitochondrial DNA [mtDNA] copy number). For HbA1c, we referred to the randomized controlled trial by Usui et al. (13), which reported mean reductions at 24 weeks of −0.75 ± 0.12% for imeglimin and −0.83 ± 0.18% for metformin compared to baseline, with further reduction from week 12 to week 24 observed only in the imeglimin group (13). Using the pooled standard deviation from these results (≈0.15–0.18%), the corresponding Cohen’s f was estimated at ≈0.36. For mtDNA copy number, we referred to Lee et al. (2022), which showed that pharmacological intervention in T2DM patients significantly reduced mtND-1 and mtCOX-3 copy numbers compared to pre-treatment values, with effect sizes in the moderate range (Cohen’s f ≈ 0.30) (14).

Sample size was calculated using G*Power (version 3.1.9.7) for a one-way ANOVA (fixed effects, omnibus) with four groups, assuming Cohen’s f=0.36, two-sided α=0.05, and power 1−β=0.80. Cohen’s f is defined as f=σ_m_/σ_f_, where σ_m_ ​is the standard deviation of the group means and σ is the common within-group standard deviation, representing the standardized measure of between-group variance. The non-centrality parameter is λ=n·k·f^2^ with k=4 (number of groups) and f=0.36, λ=0.5184n. Degrees of freedom: df1=k−1=3, df2=k(n−1). Power was computed from the non-central F-distribution using


$$\text{Power}=1-{\text{F}}_{\text{nc}-\text{CDF}\;}\left(F_{0.95}\left(3,4\left(\text{n}-1\right)\right);3,4\left(\text{n}-1\right),\text{λ}\right),$$

where F_nc-CDF_ is the cumulative distribution function of the non-central F-distribution. G*Power iteratively increases n until the computed power meets or exceeds the target (1−β=0.80). Solving gives n=22 per arm (N=88 total).

### 2.2 Ethical considerations

Written informed consent was obtained from all participants prior to enrollment. The study protocol was approved by the Institutional Ethics Committee (IEC) of SRM Medical College Hospital and Research Centre. (IEC No: 8708/IEC/2023) and registered with Clinical Trial Registry – India (CTRI Registration No: CTRI/2023/12/060844).

### 2.3 Inclusion and exclusion criteria

Inclusion criteria included adult patients (aged 30–70 years) diagnosed with T2DM according to ADA 2024 criteria, HbA1c levels between 7% and 10%, stable body weight (±2 kg) in the past three months, and willingness to adhere to study visits.

Exclusion criteria comprised patients with type 1 diabetes, recent infections, surgeries, autoimmune or chronic inflammatory disorders, malignancy, use of corticosteroids or immunosuppressants, liver dysfunction (ALT or AST >3× ULN), or chronic kidney disease stage 4 or higher (eGFR<30 mL/min/1.73 m²).

### 2.4 Study groups and treatment allocation

Participants were assigned into four treatment groups (minimum n=22 per group), based on their current or newly initiated therapy:

1. Group A: Imeglimin Monotherapy
2. Group B: Imeglimin + Metformin
3. Group C: Imeglimin + Other Oral Hypoglycemic Agents (OHAs)
4. Group D: Metformin + Other OHAs (Control Group)

Treatment regimens were maintained under routine clinical care and supervised follow-up throughout the study period.

Participants received Imeglimin orally at 1,000 mg twice daily (totaling 2,000 mg per day), in accordance with standard dosing recommendations. Metformin was given at conventional doses ranging from 500 mg to 2,000 mg daily, administered once or twice per day depending on individual glycemic targets and tolerability. All medications were prescribed following standard clinical guidelines.

This was a non-randomized, non-blinded clinical trial. Group allocation was determined pragmatically based on participants’ treatment history and clinical requirements, rather than random assignment. Patients in the Imeglimin monotherapy group were treatment-naïve and received Imeglimin as their initial anti-diabetic therapy. For combination therapy groups, Imeglimin was added to pre-existing anti-diabetic regimens in patients with suboptimal glycemic control. Specifically, the Imeglimin + Metformin and Imeglimin + other oral hypoglycemic agent (OHA) groups included patients whose HbA1c remained above target despite ongoing therapy, prompting the addition of Imeglimin. The Metformin + other OHA group consisted of individuals continuing Metformin alongside other agents, such as sulfonylureas, DPP-4 inhibitors, or SGLT2 inhibitors. Medication adherence was regularly monitored throughout the study period.

### 2.5 Data collection and follow-up

Participants underwent detailed clinical and biochemical evaluation at baseline and at 6-month follow-up. Clinical data included age, gender, body mass index (BMI), duration of diabetes, and blood pressure. Laboratory parameters included fasting plasma glucose, HbA1c, lipid profile, and serum creatinine.

### 2.6 Biomarker assessment

#### 2.6.1 Quantification of circulating cell-free mitochondrial DNA

Peripheral venous blood was collected from participants in sterile serum separator tubes following an overnight fast, at both the initial and 6-month visits. After centrifugation at 3000 rpm for 10 minutes at 4°C, serum was isolated and stored at −80°C. Cell-free DNA was extracted from 200 µL of serum using the QIAamp Circulating Nucleic Acid Kit (Qiagen, Germany) as per the manufacturer’s instructions, and the eluted DNA was stored at −20°C until PCR analysis.

Quantification of circulating cell-free mitochondrial DNA (ccf-mtDNA) was performed via real-time quantitative PCR (qPCR) using the ABI PRISM 7900HT System (Applied Biosystems, USA). The mitochondrial ND1 gene was amplified using specific primers (forward: 5’-CCCTAAAACCCGCCACATCT-3’; reverse: 5’-GAGCGATGGTGAGAGCTAAGGT-3’), while β-globin primers served as a nuclear DNA control (forward: 5’-GTGCACCTGACTCCTGAGGAGA-3’; reverse: 5’-CCTTGATACCAACCTGCCCAG-3’). PCR reactions (20 µL total volume) included SYBR Green Master Mix, primers, template DNA, and nuclease-free water. Thermal cycling involved denaturation at 95°C for 10 minutes, followed by 40 cycles of 95°C for 15 seconds and 60°C for 60 seconds. Melt curve analysis was conducted to confirm amplification specificity. Relative ccf-mtDNA levels were determined using the ΔCt method (Ct_nuclear – Ct_mitochondrial), where lower ΔCt values indicate higher mtDNA concentration (15).

#### 2.6.2 Serum NLRP3 inflammasome quantification

Serum NLRP3 concentrations were measured using a human-specific sandwich ELISA kit (Elabscience Biotechnology Inc., USA). All reagents and serum samples were equilibrated to room temperature before use. Standards and diluted serum samples (100 µL) were added in duplicate to a 96-well plate pre-coated with anti-NLRP3 antibodies and incubated at 37°C for 90 minutes. After washing to remove unbound material, 100 µL of biotinylated detection antibody was added and incubated for 1 hour, followed by HRP-conjugated streptavidin for 30 minutes.

Color development was performed using TMB substrate and stopped upon sufficient signal intensity. Absorbance was read at 450 nm using a microplate reader. The assay had a sensitivity of<9.38 pg/mL and a detection range of 15.6–1000 pg/mL. Intra- and inter-assay coefficient of variation (CV) values were<10% and<12%, respectively. Final concentrations were determined using a standard curve derived from recombinant NLRP3 protein.

#### 2.6.3 Inflammatory markers: IL-18, IL-1β and IL-6 measurement

Serum concentrations of Interleukin-18 (IL-18), Interleukin-1β (IL-1β) and Interleukin-6 (IL-6) were determined using high-sensitivity ELISA kits for, IL-1β and IL-6. All assays were performed as per the manufacturers’ protocols, and all samples were analyzed in duplicate. Standard curves were generated using known concentrations of recombinant cytokines and fitted using a four-parameter logistic (4-PL) model for accurate interpolation of unknown concentrations.

These inflammatory markers were selected based on their strong relevance to NLRP3-mediated inflammatory pathways and mitochondrial stress in the context of type 2 diabetes.

### 2.7 Statistical analysis

All analyses were conducted using IBM SPSS Statistics (version [insert version]). Paired t-tests were used to compare baseline and post-treatment values within groups. Repeated measures ANCOVA analysis assessed changes over time across treatment arms. Pearson or Spearman correlation analyses were performed based on data distribution to evaluate associations between variables. Multivariate analysis was conducted to adjust for potential confounders. A p-value< 0.05 was considered statistically significant.

## 3 Results

### 3.1 Baseline characteristics of study groups

Baseline characteristics of the study groups are presented in **Table 1**. There were no significant differences among the Imeglimin Monotherapy, Imeglimin + Metformin, Imeglimin + Other OHAs, and Control (Metformin + Other OHAs) groups in terms of age (mean range 54.3–56.1 years, p=0.79), sex distribution (male 52.4%–58.5%, p=0.93), duration of diabetes (0.8–1.7 years, p=0.68), or BMI (25.5–26.9 kg/m²), p=0.81). Lipid profiles, including total cholesterol, LDL-C, VLDL-C, HDL-C, and triglycerides, as well as glycemic control measured by HbA1c (7.8%–8.0%, p=0.20), did not differ significantly between groups. Similarly, renal function markers (serum creatinine and UACR), circulating cell-free mitochondrial DNA, and inflammatory cytokines (NLRP3, IL-6, IL-1β, IL-18) were comparable across groups (all p > 0.05). These results demonstrate that baseline demographic and clinical parameters were well balanced, minimizing confounding in subsequent analyses. This suggests a well-matched cohort at baseline, ensuring minimal bias when comparing post-intervention outcomes. (**Table 1**). No serious adverse events occurred.

**Table 1:** Baseline characteristics of the study.

Variable	Imeglimin monotherapy (n=23)	Imeglimin + metformin (n=24)	Imeglimin + other oral hypoglycemic agents (OHAs) (n=24)	Control (metformin + + other oral hypoglycemic agents (OHAs) (n=25)	P-value
Age (years, mean ± SD)	56.1 ± 6.3	55.6 ± 7.5	54.3 ± 6.9	55.2 ± 7.1	0.79 (ANOVA)
Sex (Male, %)	57.9%	52.4%	54.2%	58.5%	0.93 (Chi-square)
Duration of Diabetes (years, mean ± SD)	0.8 ± 0.4	1.7 ± 0.2	1.4 ± 0.6	1.6 ± 0.3	0.68 (ANOVA)
BMI (kg/m², mean ± SD)	25.8 ± 2.7	26.9 ± 3.2	25.5 ± 2.4	26.6 ± 3.6	0.81 (ANOVA)
Total Cholesterol (mg/dL)	182.4 ± 28.7	176.3 ± 33.1	188.7 ± 25.6	180.9 ± 22.4	0.67 (ANOVA)
LDL-C (mg/dL)	112.3 ± 26.4	109.8 ± 24.7	114.5 ± 23.8	111.6 ± 25.9	0.88 (ANOVA)
VLDL-C (mg/dL)	26.7 ± 5.9	27.3 ± 6.0	27.0 ± 5.4	26.8 ± 6.1	0.94 (ANOVA)
HDL-C (mg/dL)	43.1 ± 6.8	42.9 ± 7.2	43.0 ± 6.7	42.1 ± 7.0	0.76 (ANOVA)
Triglycerides (mg/dL, median, IQR)	134 (119–148)	137 (124–150)	135 (122–149)	133 (121–148)	0.69 (Kruskal-Wallis)
HbA1c (%, mean ± SD)	7.8 ± 1.2	7.8 ± 0.7	7.9 ± 0.4	8.0 ± 1.4	0.20 (ANOVA)
Serum Creatinine (mg/dL)	0.91 ± 0.16	0.94 ± 0.15	0.90 ± 0.13	0.92 ± 0.17	0.72 (ANOVA)
UACR (mg/g, median, IQR)	34 (26–48)	36 (28–51)	35 (27–50)	37 (26–53)	0.61 (Kruskal-Wallis)
ccf-mtDNA (copies/μL, mean ± SD)	34.5 ± 1.2	32.1 ± 1.0	34.3 ± 1.1	35.0 ± 1.3	0.47 (ANOVA)
Serum NLRP3 (pg/mL, mean ± SD)	162.3 ± 28.5	158.9 ± 25.1	160.1 ± 27.6	174.2 ± 30.4	0.39 (ANOVA)
IL-6 (pg/mL, mean ± SD)	4.3 ± 0.8	4.4 ± 0.9	4.2 ± 0.7	4.1 ± 0.8	0.68 (ANOVA)
IL-1β (pg/mL, mean ± SD)	3.8 ± 0.9	3.6 ± 0.7	3.7 ± 0.8	3.9 ± 1.0	0.77 (ANOVA)
IL-18 (pg/mL, mean ± SD)	7.6 ± 1.6	7.9 ± 1.5	8.1 ± 1.6	7.8 ± 1.7	0.78 (ANOVA)

BMI, Body Mass Index; LDL-C, Low-Density Lipoprotein Cholesterol; VLDL-C, Very Low-Density Lipoprotein Cholesterol; HDL-C, High-Density Lipoprotein Cholesterol; HbA1c, Glycated Hemoglobin A1c; UACR, Urinary Albumin-to-Creatinine Ratio; ccf-mtDNA, Circulating Cell-Free Mitochondrial DNA; NLRP3, NOD-like Receptor Protein 3 and IL, Interleukin. Statistical analyses were performed using ANOVA (Analysis of Variance) for comparisons of means between groups, Chi-square test for comparisons of proportions, and Kruskal-Wallis test for comparisons of medians. p-values indicate statistical significance, with values less than 0.05 typically considered significant.

**Figure 1:**
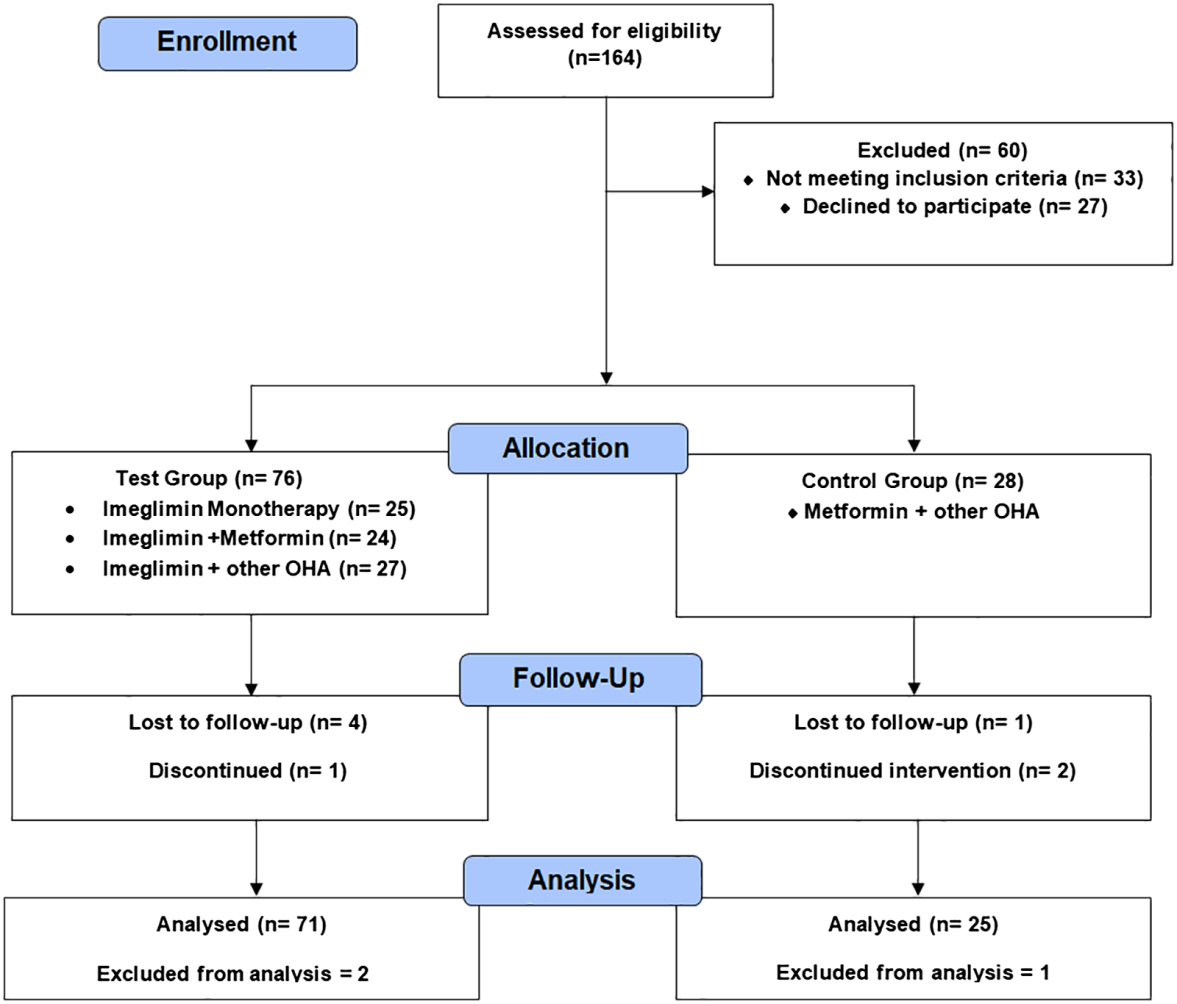
Study flow diagram of participant enrollment, grouping, follow-up, and analysis.

**Figure 1** outlines the progression of participants through the prospective observational study. A total of 164 individuals were assessed for eligibility, of whom 60 were excluded. The remaining 104 participants were grouped into a test group (Imeglimin-based therapies) and a control group (Metformin + other oral hypoglycemic agents). Follow-up was conducted at 6 months, with documentation of dropouts and discontinuations. The final analysis included 71 participants in the test group and 25 in the control group after accounting for exclusions.

### 3.2 Correlations between ccf-mtDNA and metabolic/inflammatory markers

Positive correlations were observed between Δ ccf-mtDNA and Δ HbA1c (r=0.29, p=0.005), Δ LDL-C (r=0.22, p=0.034), Δ triglycerides (r=0.25, p=0.012), and Δ IL-6 (r=0.33, p=0.002). Similarly, Δ serum NLRP3 showed significant positive correlations with Δ HbA1c (r=0.24, p=0.018), Δ LDL-C (r=0.20, p=0.047), Δ triglycerides (r=0.21, p=0.036), and Δ IL-1β (r=0.35, p=0.012). Correlations with age were mild and reached statistical significance for ccf-mtDNA (r=0.21, p=0.045) but not for serum NLRP3 (p=0.078). No significant correlations were noted with gender, diabetes duration, BMI, UACR, serum creatinine, or HDL-C for either ccf-mtDNA or NLRP3 (**Table 2**).

**Table 2:** Correlation analysis between ccf-mtDNA and serum NLRP3 with metabolic and inflammatory markers.

Parameter	Δ ccf-mtDNA (r, p-value)	Δ Serum NLRP3 (r, p-value)
Age	r=0.21, p=0.045	r=0.18, p=0.078
Gender	r=0.05, p=0.512	r=+0.03, p=0.673
Diabetes Duration	r=0.17, p=0.082	r=0.13, p=0.144
Δ HbA1c	r=0.29, p=0.005	r=0.24, p=0.018*
Δ Total Cholesterol	r=0.14, p=0.129	r=0.11, p=0.193
Δ LDL-C	r=0.22, p=0.034	r=0.20, p=0.047*
Δ HDL-C	r=0.09, p=0.274	r=+0.07, p=0.354
Δ Triglycerides	r=0.25, p=0.012	r=0.21, p=0.036*
Δ BMI	r=0.12, p=0.171	r=0.10, p=0.237
Δ UACR	r=-0.19, p=0.063	r=0.16, p=0.093
Δ Serum Creatinine	r=-0.11, p=0.201	r=0.09, p=0.268
Δ IL-6	r=0.33, p=0.002	r=0.41, p=0.131
Δ IL-18	r=0.28, p=0.087	r=0.37, p=0.14
Δ IL-1β	r=0.30, p=0.14	r=0.35, p=0.012*

Δ indicates the change in the respective parameter. Correlation coefficients (r) are provided with corresponding p-values for each parameter. Statistically significant values are marked where p< 0.05. (*p< 0.05).

Of note, although Δ serum NLRP3 had moderate correlations with Δ IL-6 (r=0.41) and Δ IL-18 (r=0.37), these did not reach statistical significance (p=0.131 and 0.14, respectively).

### 3.3 Within group comparisons of changes in ccf-mtDNA and inflammatory markers

The paired t-test analysis highlights that combination therapies, especially Imeglimin + other OHA and Metformin + other OHA, produced significant reductions in key metabolic and inflammatory parameters. HbA1c levels significantly decreased in the Imeglimin + other OHA group (Δ=-0.5%, p=0.001) and in the Metformin + other OHA group (Δ=-0.3%, p=0.002), indicating improved glycemic control with these regimens. In contrast, Imeglimin monotherapy and its combination with Metformin did not achieve statistically significant HbA1c reduction.

Importantly, circulating cell-free mitochondrial DNA (ccf-mtDNA), a marker of mitochondrial stress, was significantly reduced in both the Imeglimin + other OHA (Δ=-18.5 copies/μL, p<0.001) and Metformin + other OHA groups (Δ=-9.4 copies/μL, p=0.002). Regarding inflammation, significant reductions in IL-6 were observed across all combination groups, with the greatest effect in the Imeglimin + other OHA group (Δ=-1.5 pg/mL, p< 0.001). IL-1β also decreased significantly in the Imeglimin + Metformin and Imeglimin + other OHA groups. However, changes in NLRP3 levels did not reach statistical significance, though downward trends were noted. These findings suggest that Imeglimin, particularly in combination with other agents, may improve glycemic regulation and mitigate mitochondrial and inflammatory stress in patients. (**Table 3**).

**Table 3:** Paired t-test analysis for change in parameters after 6 months of intervention.

Parameter	Group	Mean difference (Δ) ± SD	t-value	p-value	95% CI for Δ
HbA1c (%)	Imeglimin monotherapy	-0.2 ± 0.7	-2.21	0.03*	(-0.38, 0.02)
	Imeglimin + Metformin	-0.3 ± 0.2	-2.33	0.024*	(-0.76, 0.06)
	Imeglimin + other OHA	-0.5 ± 0.6	-3.5	0.001**	(-0.75, -0.25)
	Metformin + other OHA	-0.3 ± 0.5	-3.2	0.002*	(-0.65, -0.15)
BMI (kg/m²)	Imeglimin monotherapy	-0.1 ± 0.6	-0.8	0.43	(-0.40, 0.18)
	Imeglimin + Metformin	-0.2 ± 0.7	-1.4	0.16	(-0.52, 0.08)
	Imeglimin + other OHA	-0.3 ± 0.6	-1.8	0.07	(-0.62, 0.02)
	Metformin + other OHA	-0.2 ± 0.6	-1.3	0.21	(-0.50, 0.10)
Triglycerides (mg/dL)	Imeglimin monotherapy	-9.5 ± 24.2	-1.7	0.09	(-20.5, 1.5)
	Imeglimin + Metformin	-11.0 ± 26.1	-1.9	0.06	(-22.5, 0.5)
	Imeglimin + other OHA	-8.2 ± 21.5	-1.5	0.13	(-19.0, 2.6)
	Metformin + other OHA	-6.8 ± 22.7	-1.4	0.18	(-17.7, 4.1)
UACR (mg/g)	Imeglimin monotherapy	-1.5 ± 6.3	-1.3	0.19	(-3.7, 0.7)
	Imeglimin + Metformin	-3.1 ± 8.2	-1.8	0.08	(-6.5, 0.3)
	Imeglimin + other OHA	-2.8 ± 9.0	-1.4	0.17	(-6.3, 0.7)
	Metformin + other OHA	-1.7 ± 7.5	-1.2	0.23	(-4.5, 1.1)
ccf-mtDNA (copies/μL)	Imeglimin monotherapy	-6.2 ± 1.2	-2.88	0.064	(-13.3, 0.9)
	Imeglimin + Metformin	-4.7 ± 1.01	-6.38	0.002*	(-6.9, -2.5)
	Imeglimin + other OHA	-18.5 ± 3.2	-6.23	0.0003*	(-22.0, -15.0)
	Metformin + other OHA	-9.4 ± 1.9	-5.23	0.002*	(-12.8, -6.0)
NLRP3 (ng/mL)	Imeglimin monotherapy	6.3 ± 1.8	-1.3	0.064	(-1.2, 0.2)
	Imeglimin + Metformin	-5.4 ± 2.0	-1.6	0.058	(-1.8, 0.2)
	Imeglimin + other OHA	-7.9 ± 1.6	-1.5	0.042*	(-1.5, 0.2)
	Metformin + other OHA	-10.6 ± 1.1	-1.4	0.073	(-1.4, 0.2)
IL-6 (pg/mL)	Imeglimin monotherapy	-0.4 ± 0.9	-2.1	0.041	(-0.8, -0.02)
	Imeglimin + Metformin	-0.7 ± 1.1	-3.2	0.002*	(-1.2, -0.3)
	Imeglimin + other OHA	-1.5 ± 1.2	-5.4	<0.001**	(-2.0, -1.0)
	Metformin + other OHA	-1.1 ± 1.3	-4.3	<0.001**	(-1.6, -0.6)
IL-18 (pg/mL)	Imeglimin monotherapy	-0.7 ± 1.7	-1.2	0.24	(-2.1, 0.7)
	Imeglimin + Metformin	-1.0 ± 1.5	-1.7	0.10	(-2.2, 0.2)
	Imeglimin + other OHA	-1.1 ± 1.6	-1.8	0.08	(-2.4, 0.2)
	Metformin + other OHA	-0.6 ± 1.8	-1.1	0.28	(-2.1, 0.9)
IL-1β (pg/mL)	Imeglimin monotherapy	-0.5 ± 1.2	-1.3	0.19	(-1.3, 0.3)
	Imeglimin + Metformin	-0.8 ± 0.9	-2.4	0.022*	(-1.4, -0.1)
	Imeglimin + other OHA	-1.0 ± 1.0	-2.8	0.008*	(-1.7, -0.3)
	Metformin + other OHA	-0.6 ± 1.1	-1.6	0.13	(-1.4, 0.2)

Paired t-test statistics include mean difference (Δ) ± standard deviation (SD), t-value, degrees of freedom (df), and p-value. The 95% confidence interval (CI) for the mean difference is also provided. Statistically significant results are marked where p< 0.05. (*p< 0.05; **p< 0.001).

### 3.4 Between-group comparison of treatment effects on HbA1c, ccf-mtDNA, and serum NLRP3 levels using repeated measures ANCOVA

Repeated measures ANCOVA revealed significant time effects for all three parameters, indicating overall changes from baseline to 6 months across treatment groups (**Table 4**). Specifically, HbA1c levels decreased significantly over time (F=12.4, p<0.001), with the greatest reduction observed in the Imeglimin + Other OHAs group compared to control (p=0.002). The group effect was also significant (F=3.6, p=0.015), and a significant time-by-group interaction (F=4.2, p=0.008) indicated differential treatment responses over time. Notably, Imeglimin + Metformin showed a trend toward HbA1c reduction versus control (p=0.053), while Imeglimin monotherapy did not differ significantly from control.

**Table 4:** Adjusted changes in HbA1c, ccf-mtDNA, and serum NLRP3 levels at 6 months by treatment group (RM-ANCOVA analysis).

Parameter	Effect	F-value	P-value	*Post Hoc* pairwise comparisons at 6 months (vs. control group)
HbA1c (%)	Time	12.4	<0.001**	• Imeglimin Monotherapy vs Control: p=0.15 (no significant change)
	Group	3.6	0.015*	• Imeglimin + Metformin vs Control: p=0.053 (trend towards reduction)
	Time × Group	4.2	0.008*	• Imeglimin + Other OHAs vs Control: p=0.002 (significant reduction)
ccf-mtDNA (copies/μL)	Time	17.6	<0.001**	• Imeglimin Monotherapy vs Control: p=0.06 (trend towards reduction)
	Group	3.3	0.030*	• Imeglimin + Metformin vs Control: p=0.015 (significant reduction)
	Time × Group	4.1	0.009*	• Imeglimin + Other OHAs vs Control: p=0.020 (significant reduction)
Serum NLRP3 (ng/mL)	Time	6.5	0.012*	• Imeglimin Monotherapy vs Control: p=0.064 (no significant change)
	Group	1.2	0.310	• Imeglimin + Metformin vs Control: p=0.058 (trend towards reduction)
	Time × Group	2.7	0.060	• Imeglimin + Other OHAs vs Control: p=0.042 (significant reduction)

Statistically significant results are marked where p< 0.05. (*p< 0.05; **p< 0.001).

For circulating cell-free mitochondrial DNA (ccf-mtDNA), significant time (F=17.6, p< 0.001), group (F=3.3, p=0.030), and time-by-group interaction effects (F=4.1, p=0.009) were observed. *Post hoc* analysis demonstrated significant reductions in ccf-mtDNA at 6 months in the Imeglimin + Other OHAs group (p=0.020) and the Imeglimin + Metformin group (p=0.015) compared to the control group. A non-significant trend toward reduction was also observed in the Imeglimin Monotherapy group (p=0.06).

Serum NLRP3 levels also showed a significant time effect (F=6.5, p=0.012), with a trend toward group differences (F=1.2, p=0.31) and a borderline time-by-group interaction (F=2.7, p=0.06). *Post hoc* comparisons revealed a significant decrease in NLRP3 levels with Imeglimin + Other OHAs relative to control (p=0.042), while reductions in other groups were non-significant or trending.

Overall, these findings suggest that combination therapies involving Imeglimin, particularly with other OHAs, produce more robust improvements in glycemic control, mitochondrial DNA release, and inflammasome activity compared to control therapy over 6 months.

### 3.5 Effect of Imeglimin-based therapies on serum interleukin levels over time

The repeated measures ANOVA revealed significant time-dependent reductions in inflammatory cytokines, particularly IL-6 and IL-1β, among patients receiving combination therapies (**Table 5**). IL-6 levels showed a robust time effect (F=20.8, p< 0.001) with a significant time × group interaction (F=6.8, p< 0.001). *Post hoc* comparisons indicated a marked reduction in IL-6 at 6 months in the Imeglimin + other OHA group (p=0.002) and the Imeglimin + Metformin group (p=0.007), whereas Imeglimin monotherapy did not differ significantly from controls (p=0.45).

**Table 5:** Effect of Imeglimin-based therapies on inflammatory cytokines over time.

Parameter	Effect	F-value	P-value	*Post Hoc* pairwise comparisons at 6 months (vs. control group)
IL-6	Time	20.8	<0.001**	Imeglimin + Other Oral Hypoglycemic Agents vs Control: p=0.002 (significant reduction) Imeglimin + Metformin vs Control: p=0.007 (significant reduction) Imeglimin Monotherapy vs Control: p=0.45 (no significant change)
	Group	1.1	0.35
	Time × Group	6.8	<0.001**
IL-18	Time	1.8	0.18	No significant pairwise differences
	Group	0.9	0.44	
	Time × Group	0.8	0.50	
IL-1β	Time	7.5	0.007*
	Group	1.0	0.39
	Time × Group	3.3	0.022*	Imeglimin + Other Oral Hypoglycemic Agents vs Control: p=0.045 (significant reduction) Imeglimin Monotherapy vs Control: p=0.15 (no significant change) Imeglimin + Metformin vs Control: p=0.12 (no significant change)

Statistically significant results are marked where p< 0.05. (*p< 0.05; **p< 0.001).

For IL-1β, a significant time effect (F=7.5, p=0.007) and a time × group interaction (F=3.3, p=0.022) were observed. A significant reduction was evident only in the Imeglimin + other OHA group when compared with controls (p=0.045). No statistically meaningful changes were found in IL-18 levels across any treatment arms. These findings underscore the anti-inflammatory potential of Imeglimin when used in combination regimens, particularly with other oral hypoglycemic agents.

## 4 Discussion

In this study, baseline characteristics were well-matched across the four intervention groups, ensuring comparability for evaluating the effects of Imeglimin and combination therapies. Our findings demonstrated that combination therapies involving Imeglimin or Metformin with other oral hypoglycemic agents (OHAs) led to significant improvements in glycemic control, as evidenced by reductions in HbA1c, whereas Imeglimin monotherapy and the Imeglimin + Metformin group did not achieve statistically significant changes. Singh et al. found that imeglimin (1000 mg BID) effectively reduced HbA1c in T2D patients across Phase 2 and 3 trials. It showed comparable efficacy to metformin, SUs, AGIs, DPP-4is, and SGLT-2is, but lower than TZDs. Combination therapy with metformin and TZDs enhanced HbA1c reduction, whereas no significant benefit was observed with GLP-1RAs. Imeglimin demonstrated good tolerability, fewer gastrointestinal side effects than metformin, and no cases of lactic acidosis, suggesting a favorable safety and efficacy profile (16). The INDI-TIMES study, India’s largest real-world analysis of imeglimin in T2DM (n=8301), showed that imeglimin 1000 mg twice daily significantly reduced HbA1c (−1.12%), FPG, PPG, improved lipid profiles, and lowered serum creatinine. Results were consistent across monotherapy and combination therapy groups. Compared to TIMES studies in Japanese patients, a greater HbA1c reduction was seen, possibly due to higher baseline glycemia in Indian patients. Weight loss and improved liver enzymes were also observed without major safety concerns, supporting imeglimin’s effectiveness in routine clinical practice (17). Imeglimin add-on therapy to metformin acutely reduced 24-hour mean glucose levels and improved glycemic variability in patients with type 2 diabetes. Higher baseline HDL cholesterol levels correlated with greater improvements. Continuous glucose monitoring confirmed significant increases in time in range and decreases in glucose variability after imeglimin initiation (18). Yang et al. (2020) observed that metformin treatment over one year significantly reduced peripheral mitochondrial DNA copy number (mtDNA-CN) in women with polycystic ovary syndrome. The reduction in mtDNA-CN correlated with decreased serum testosterone but not with metabolic factors, suggesting a link between mitochondrial function improvement and androgen regulation (19). Wang et al. (2023) found that mitochondrial DNA copy number (mtDNA-CN) may help guide treatment choice between metformin and acarbose in newly diagnosed type 2 diabetes. Higher mtDNA-CN was associated with better β-cell function protection with metformin but worse with acarbose, although no association with glycemic response was observed (20). Liu et al. (21) demonstrated that circulating cell-free mitochondrial DNA (ccf-mtDNA) levels were significantly elevated in coronary heart disease (CHD) patients with type 2 diabetes compared to those without. Plasma ccf-mtDNA showed strong diagnostic value (AUC 0.907), and levels correlated with fasting glucose, suggesting its potential as a biomarker (21). Bae et al. (9) reported that plasma ccf-mtDNA levels were significantly elevated in patients with type 2 diabetes compared to healthy controls. Elevated ccf-mtDNA correlated with IL-1β levels and activated the AIM2 inflammasome in macrophages, suggesting that ccf-mtDNA contributed to chronic inflammation through AIM2 inflammasome-mediated pathways in type 2 diabetes (9). Previous studies have demonstrated that metformin modulates mitochondrial DNA copy number and mitochondrial function (19, 20), and that elevated circulating cell-free mitochondrial DNA is associated with diabetic complications such as coronary heart disease, considering imeglimin’s structural similarity to metformin and its targeting of mitochondrial bioenergetics, we hypothesize that imeglimin could exert a similar modulatory effect on mitochondrial health, with this study being the first to explore this possibility. This study represents the first attempt to explore this potential effect with imeglimin.

**Figure 2:**
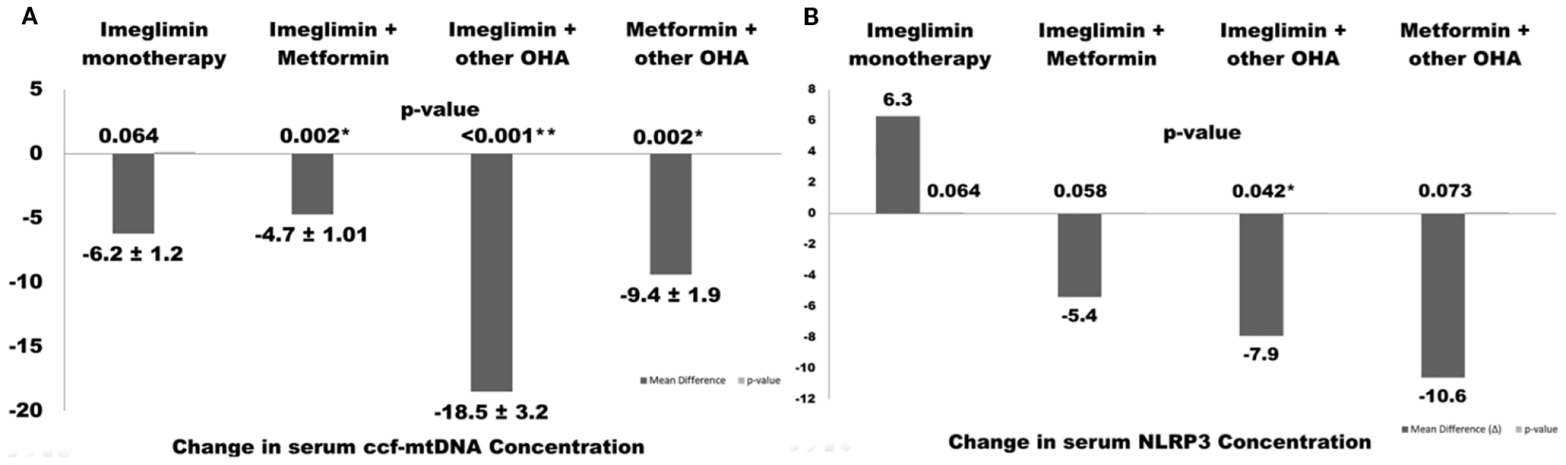
Changes in serum ccf-mtDNA **(A)** and NLRP3 **(B)** concentrations following different therapeutic regimens in type 2 diabetes patients over 6 months. Values are presented as mean ± SEM. Asterisks indicate statistically significant changes (p< 0.05).

Interestingly, significant reductions in circulating cell-free mitochondrial DNA (ccf-mtDNA) were observed in the Imeglimin + Metformin and Imeglimin + Other OHA groups, suggesting a potential synergistic effect on mitochondrial stress or turnover when Imeglimin is combined with other agents. **Figure 2** illustrates changes in serum ccf-mtDNA (A) and NLRP3 (B) concentrations across different therapeutic regimens in T2DM patients over 6 months. The combination of Imeglimin with other OHAs showed the most significant reductions in both ccf-mtDNA (–18.5 ± 3.2 copies/μL, *p* < 0.001) and NLRP3 levels (–7.9 ± 1.6 ng/mL, *p* = 0.042), indicating improved mitochondrial integrity and reduced inflammation. Metformin + other OHA also significantly reduced ccf-mtDNA (–9.4 ± 1.9, *p* = 0.002), while changes in other groups were not statistically significant. Notably, Imeglimin monotherapy led to a non-significant increase in NLRP3, suggesting limited anti-inflammatory effect when used alone (**Figure 2**).

**Figure 3:**
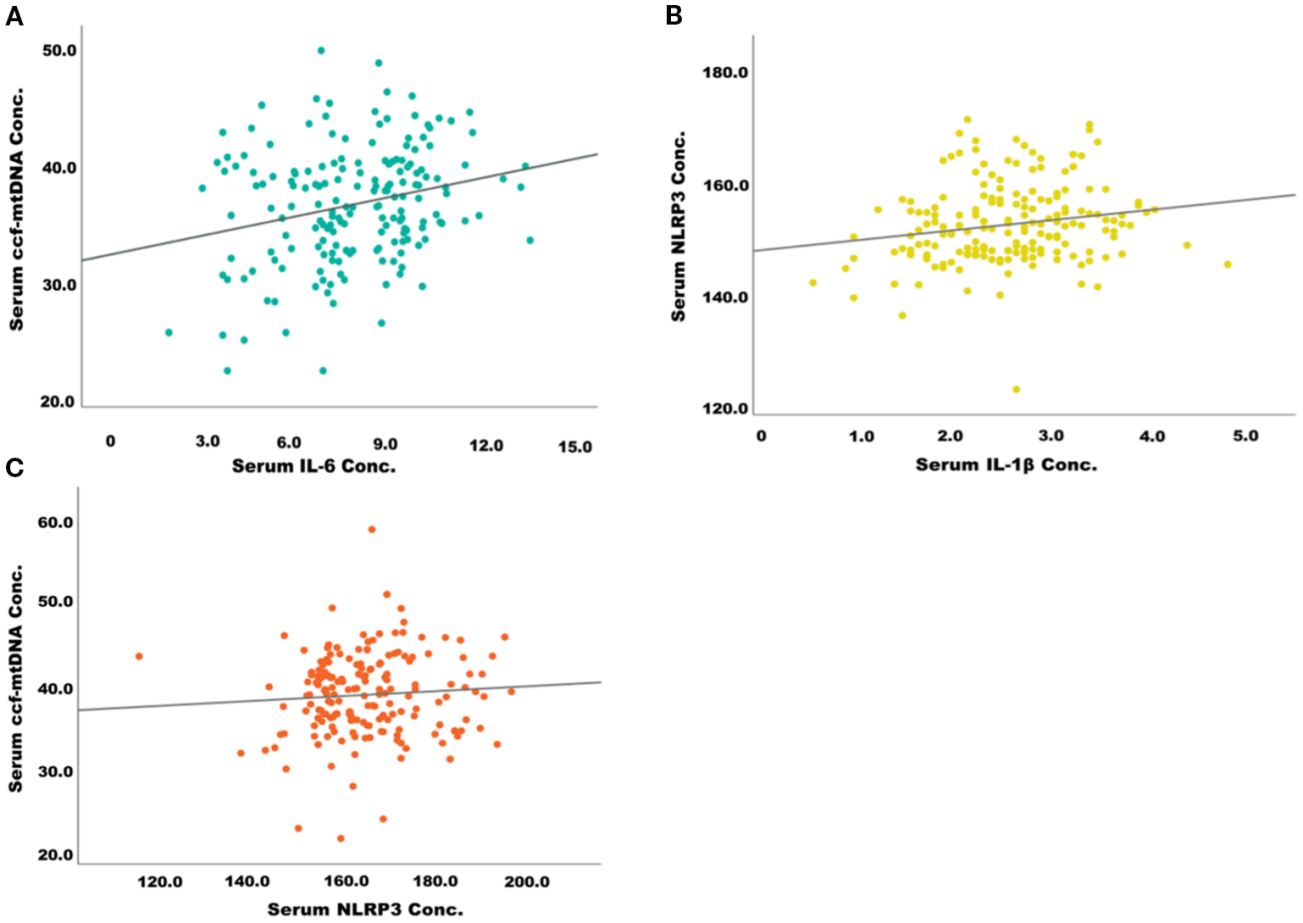
Correlation between inflammatory markers and mitochondrial stress biomarkers in patients with type 2 diabetes. **(A)** Positive correlation between serum IL-6 and ccf-mtDNA concentration. **(B)** Positive correlation between serum IL-1β and NLRP3 concentration. **(C)** Positive correlation between serum NLRP3 and ccf-mtDNA concentration.

The scatter plots in **Figure 3** illustrate significant positive correlations between markers of systemic inflammation and mitochondrial stress in patients with type 2 diabetes mellitus (T2DM). Panel 2A shows a positive association between serum IL-6 levels and circulating cell-free mitochondrial DNA (ccf-mtDNA), suggesting that increased systemic inflammation may be linked to elevated mitochondrial stress or damage. Panel 2B reveals a positive correlation between IL-1β and NLRP3 concentrations, indicating the activation of the NLRP3 inflammasome pathway in response to pro-inflammatory stimuli. Furthermore, Panel 2C demonstrates a mild positive correlation between serum NLRP3 and ccf-mtDNA concentrations, implying a potential crosstalk between mitochondrial dysfunction and innate immune activation. Collectively, these findings suggest that mitochondrial-derived danger signals, such as ccf-mtDNA, may contribute to the activation of inflammatory pathways in T2DM, potentially playing a role in the progression of metabolic inflammation. These findings imply that Imeglimin exerts optimal anti-inflammatory and mitochondrial protective effects when used in combination therapy, especially with Metformin or other OHAs. Alfadul et al. (2023) found that six months of lifestyle modification in prediabetic individuals significantly modulated serum NLRP3 inflammasome activity and related interleukins. Glycemic control led to reductions in NLRP3, IL-33, and IL-1α, suggesting that early intervention could reverse inflammatory changes and reduce progression to type 2 diabetes mellitus (22). Zhang et al. demonstrated that metformin corrected glycometabolic reprogramming and suppressed NLRP3 inflammasome-induced pyroptosis in trophoblasts by inhibiting the TLR4/NF-κB/PFKFB3 signaling pathway. Their findings suggested that metformin mitigated oxidative stress and inflammatory pyroptosis (23). Several other animal and preclinical studies have demonstrated the therapeutic effects of metformin in inhibiting NLRP3 inflammasome activation, and due to the structural and mechanistic similarities between metformin and imeglimin, it is hypothesized that imeglimin may exert a similar modulatory effect on NLRP3-mediated inflammation (24, 25). However, despite moderate correlations between inflammatory markers and metabolic parameters, In the current study no significant longitudinal changes in serum NLRP3 or other inflammatory cytokines were detected, indicating that a 6-month intervention period may be insufficient to produce measurable anti-inflammatory effects, or that mitochondrial and glycemic improvements precede detectable changes in systemic inflammation. The positive correlations between changes in ccf-mtDNA and glycemic and lipid parameters underscore the intertwined relationship between mitochondrial health and metabolic control. In contrast to our findings, Berezina et al. (2023) demonstrated that circulating cell-free mitochondrial DNA (cf-mtDNA) levels were significantly lower in T2DM patients with heart failure compared to those without. Their results suggest a depletion of cf-mtDNA in advanced cardiac dysfunction, diverging from the elevated cf-mtDNA levels observed in our cohort (26).

Although NLRP3 expression showed only a falling trend without statistical significance, reductions in IL-6 and IL-1β suggest partial suppression of inflammasome activity. Prior studies support such effects, as Kim et al. showed SGLT2 inhibition lowers IL-1β via ketone- and insulin-mediated mechanisms, and Birnbaum et al. reported SGLT2 ± DPP4 inhibition attenuates inflammasome activation and renal injury (1,2). In our cohort, “other OHAs” comprised sulfonylureas and DPP4 inhibitors, which may provide anti-inflammatory benefits. The weak correlation and modest changes observed could also be attributed to our relatively low sample size, potentially underpowering statistical significance (27, 28). Collectively, these findings highlight the greater efficacy of combination therapies in improving glycemic and mitochondrial parameters, while suggesting that more prolonged or intensified interventions may be required to influence inflammatory pathways.

In our study, the HbA1c reduction observed in the Imeglimin + OHA group (Δ=–0.5%) was lower than that reported in the INDI-TIMES study (Δ=–1.12%) (17). Several factors may explain this difference. First, the baseline HbA1c levels in our cohort were slightly lower (7.9%) compared to those in INDI-TIMES (8.07%). Since the degree of HbA1c reduction is strongly influenced by initial glycemic status, a lower starting HbA1c generally results in a smaller absolute decrease following therapy. Second, differences in background therapy composition could have influenced the glycemic effect. INDI-TIMES enrolled a heterogeneous population on a variety of oral hypoglycemic agents, including sulfonylureas, while our cohort included only patients on DPP-4 inhibitors and SGLT2 inhibitors in combination with Imeglimin. Both of these classes are associated with modest HbA1c reductions and may offer less additive benefit when combined with Imeglimin, compared to agents with stronger glucose-lowering potential such as sulfonylureas. Third, the study population size was considerably larger in INDI-TIMES (n=7821) compared to our sample, increasing the precision of effect size estimates and possibly capturing a greater range of treatment responses.

Together, these factors likely contributed to the smaller HbA1c reduction observed in our study. This highlights the importance of considering baseline glycemia, background therapy type, and study design differences when comparing across trials, and suggests that the magnitude of Imeglimin’s effect may vary according to patient and treatment characteristics.

While this study provides novel insights into the effects of Imeglimin on ccf-mtDNA and NLRP3 inflammasome activity in T2DM, several limitations should be noted. The relatively small sample size limits the statistical power and generalizability of our findings, and therefore the results should be interpreted in an explorative context. The 6-month duration may be insufficient to capture long-term effects, and the modest sample size limits the power for subgroup analyses. The study focused on circulating biomarkers without assessing tissue-specific or functional mitochondrial parameters. Future research should involve longer follow-up, larger and more diverse cohorts, and incorporation of functional mitochondrial assessments. Exploring tissue-specific pathways and combining Imeglimin with lifestyle or adjunctive therapies could further clarify its therapeutic potential in T2DM management. Although preclinical evidence suggests that Imeglimin, similar to metformin, may modulate NLRP3 inflammasome activity, our clinical findings (greater effectiveness with Imeglimin plus other OHAs compared to Imeglimin plus metformin) do not directly support this hypothesis. The efficacy against NLRP3 may also vary depending on the specific combination drug used with Imeglimin, which could influence the overall anti-inflammatory effect. This highlights the need for further mechanistic studies to clarify Imeglimin’s role in NLRP3-mediated inflammation and pyroptosis.

## 5 Conclusion

Imeglimin-based therapies, particularly in combination with non-metformin oral hypoglycemic agents, demonstrate significant benefits in glycemic control and may alleviate mitochondrial stress by reducing ccf mtDNA. However, direct assessment of mitochondrial function was not performed, which represents a limitation of using surrogate biomarkers. Although NLRP3 inflammasome levels did not show consistent reductions, positive correlations with glycemic and lipid changes suggest an underlying inflammatory component influenced by therapy. The findings support the use of imeglimin combinations over monotherapy for enhanced metabolic outcomes in type 2 diabetes mellitus. Further long-term studies are needed to confirm these observations and explore the mechanistic pathways linking mitochondrial function, inflammation, and glycemic control in patients undergoing imeglimin-based interventions.

## Data Availability

The original contributions presented in the study are included in the article/supplementary material. Further inquiries can be directed to the corresponding author.
